# Bio-Inspired Salinity-Gradient Power Generation With UiO-66-NH_2_ Metal-Organic Framework Based Composite Membrane

**DOI:** 10.3389/fbioe.2022.901507

**Published:** 2022-04-21

**Authors:** Lu Yao, Qi Li, Shangfa Pan, Junmei Cheng, Xueli Liu

**Affiliations:** ^1^ Key Laboratory of Rubber-Plastics, Ministry of Education, Qingdao University of Science and Technology, Qingdao, China; ^2^ Qingdao Institute of Bioenergy and Bioprocess Technology, Chinese Academy of Sciences, Qingdao, China; ^3^ College of Materials Science and Engineering, Institute of Marine Biobased Materials, Qingdao University, Qingdao, China

**Keywords:** biomimetics, energy conversion, salinity gradient, nanofluidic, metal-organic frameworks, ion transport

## Abstract

Salinity-gradient directed osmotic energy between seawater and river water has been widely considered as a promising clean and renewable energy source, as there are numerous river estuaries on our planet. In the past few decades, reverse electrodialysis (RED) technique based on cation-selective membranes has been used as the key strategy to convert osmotic energy into electricity. From this aspect, developing high-efficiency anion-selective membranes will also have great potential for capturing osmotic energy, however, remains systematically unexplored. In nature, electric eels can produce electricity from ionic gradients by using their “sub-nanoscale” protein ion channels to transport ions selectively. Inspired by this, here we developed a UiO-66-NH_2_ metal-organic framework (MOF) based anion-selective composite membrane with sub-nanochannels, and achieved high-performance salinity-gradient power generation by mixing artificial seawater (0.5 M NaCl) and river water (0.01 M NaCl). The UiO-66-NH_2_ metal-organic framework based composite membranes can be easily and economically fabricated with dense structure and long-term working stability in saline, and its performance of power generation can also be adjusted by pH to enhance the surface charge density of the MOF sub-nanochannels. This study will inspire the exploitation of MOFs for investigating the sub-nanochannel directed high-performance salinity-gradient energy harvesting systems based on anion-selective ion transport.

## Introduction

Due to the serious shortage and pollution of traditional energy sources and the increasing human demand for energy, the development of sustainable, abundant, and clean sources of energy is urgent for both the environment and human society (van Ruijven et al., 2019; Sadeghi, 2022). In the past few decades, salinity-gradient generated osmotic energy, which can be derived from ambient environments by mixing river water with salty seawater, has been recognized as a sustainable source of “blue energy” (Yip et al., 2016). Various efforts have been focusing on the development of highly-efficient salinity-gradient osmotic energy harvesting systems (Zhang et al., 2015; Xiao et al., 2019; Tawalbeh et al., 2021). Among these systems, reverse electrodialysis (RED) has been widely studied because of that electricity could be generated directly through ion transport driven by salinity gradients (Siria et al., 2017; Liu et al., 2020). An important component of the RED system is ion-selective membranes, as the permselectivity of membrane directly determines the energy conversion performance. Till now, extensive studies have been conducted, focusing on the high permselectivity of cation-selective membranes in RED systems (Zhang et al., 2015; Zhang et al., 2017; Xiao et al., 2019; Xin et al., 2019; Xin et al., 2020; Man et al., 2021). However, little research has been made on anion-selective membranes, which is to say, ignoring the possibility of energy generation with anion gradients. We consider that the use of anion-selective membranes could also be an efficient approach for salinity-gradient osmotic energy harvesting.

For achieving a high efficiency during the RED based power generation, people have learned a lot from the nature. The highly-selective ion transport thorough the sub-nanochannels of transmembrane proteins is one of the essential and fundamental activity for almost all life processes of living species (Montenegro et al., 2013; Gao et al., 2017; Ren et al., 2019; Xiao et al., 2019). For example, electric eels can produce high-voltage electricity from ionic gradients by using their “sub-nanoscale” protein ion channels (Schroeder et al., 2017; Liu et al., 2021). Inspired by that, artificial nanofluidic ion channels have been extensively investigated for their potential applications in energy conversion (Zhang et al., 2015; Gao et al., 2017; Xiao et al., 2019). Because of the unique nanoconfinement effect, the ion transport in nanofluidic channels is largely governed by the surface properties of channel walls, leading to excellent ion selectivity and high ionic throughput (Sun et al., 2020; Teng et al., 2021; Yang et al., 2021). Therefore, a variety of nanofluidic RED systems have been proposed for salinity-gradient osmotic energy harvesting. Firstly, one-dimensional (1D) single-nanopore and multi-nanopore based ion-selective membranes have been developed for the capture of osmotic energy (Gao et al., 2019; Xiao et al., 2019). However, the scalability of these nanochannels or nanopores is very hard to realize for the further commercialization, making these systems more suitable for fundamental research. As the research continues, nanofluidic heterogeneous membranes have shown their advantage for improving the power generation efficiency, thanks to their unique ionic diode effect to rectify ion transport and prohibit the flow back of current (Gao et al., 2014; Zhang et al., 2015). In addition, two-dimensional (2D) nanofluidic systems, mainly based on the stacking of 2D nanomaterials such as graphene and MXene, have also been exploited during the recent years (Zhao et al., 2015; Lao et al., 2018; Zhang et al., 2019; Ding et al., 2020; Lao et al., 2020), showing their potential in the facile fabrication of high-efficiency osmotic energy devices. However, despite of the prosperous study of nanochannel and nanopore based nanofludic RED system, ion-selective membranes based on sub-nanometer channels remain systematically unexplored for salinity-gradient osmotic energy generation, although it is sub-nanometer ion channels that are used in nature for ion transport and highly-efficient life processes.

In the respect of material selection for ion-selective membrane with sub-nanometer channels, metal-organic frameworks (MOFs) have shown their potential usage as MOFs owns three-dimensional and interconnected sub-nm-sized channels. Through the combination of variable metal clusters and ligands, MOFs have been applied to various fields such as catalysis, sensing and gas storage, thanks to their highly ordered porosity, high surface area and adjustable surface properties (Kadhom and Deng, 2018; Kirchon et al., 2018; Zhao et al., 2020; Cai et al., 2021). Recently, MOF based ion-selective membranes have also been explored for RED osmotic energy harvesting (Rice et al., 2019; Jiang et al., 2020; Tan et al., 2021). The sub-nm-sized three-dimensional interconnected channels of MOFs can provide more rigid nanoconfinements than conventional nanochannel membranes, allowing the possibility for faster and more efficient selective ion transport. This property offers new opportunities for manufacturing high-performance salinity-gradient osmotic energy generation (Lu et al., 2021), however, has not been systematically investigated till now. Particularly, UiO-66-based MOFs with tailorable surface chemistry have recently been used for ion transport (Wan et al., 2017; Li et al., 2019; Ruan et al., 2021). The channels of UiO-66-based MOFs comprise angstrom-sized windows and nm-sized cavities that comparable to most hydrated ions in water, showing great potential for highly efficient harvesting of osmotic energy. For example, a UiO-66-NH_2_ MOF based heterogeneous membrane have been fabricated for highly selective anion ion transport, and achieved highly efficient osmotic power generation under a 100-fold KBr gradient (Liu et al., 2021). Therefore, it is worthful to further explore the potential usage of UiO-66-NH_2_ MOFs as anion-selective membrane toward high-performance salinity-gradient power generator as well as other applications.

Here, based on the previous study and inspired by the sub-nanochannel based ion-transport of living systems in nature, we report an UiO-66-NH_2_ based composite membranes fabricated by a secondary-growth approach using porous anodic aluminum oxide (AAO) support, and achieved efficient anion-selective salinity-gradient power generation. The channels of the prepared UiO-66-NH_2_ MOF comprise sub-1-nanometer sized windows and nm-sized cavities with a positive surface charge because of the NH_2_ functional group. The thickness of a UiO-66-NH_2_ layer is of the submicron scale (∼710 nm). These characteristics allow the UiO-66-NH_2_ membranes to achieve rapid ion transport with low fluid resistance. The proposed UiO-66-NH_2_ membranes can achieve a maximum power density up to 1.47 W/m^2^ under 50-fold sodium chloride (NaCl) gradient (0.5 M/0.01 M), which is higher than those produced by typical commercial membranes. This UiO-66-NH_2_ based composite membranes was fabricated economically and simply without complex synthesis and expensive scientific equipment. Moreover, the membrane kept their continuous and dense structures after immersion in deionized water for 1 month, and their power density exhibited no obvious change within 1 week. Therefore, we considered that the membranes showed long-term stability. The current work can inspire research for designing anion-selective MOF based membranes, to provide more possibilities for realizing high-performance salinity-gradient osmotic power harvesting systems.

## Materials and Methods

### Materials

Zirconium (IV) chloride (ZrCl_4_), 2-aminoterephthalic acid (BDC-NH_2_), dimethylformamide (DMF), and sodium chloride (NaCl) were purchased from Sigma-Aldrich (Shanghai, China). Highly ordered porous AAO membranes (160–200 nm) were obtained from Puyuan nano (Anhui, China).

### Preparation of Single-Growth UiO-66-NH_2_ Composite Membrane

For preparing single-growth UiO-66-NH_2_ membrane, 0.116 g of ZrCl_4_ and 0.0906 g of BDC-NH_2_ were firstly ultrasonically dissolved in 30 mL of DMF, and then the resulting solution was transferred into a 50 mL Teflon-lined stainless-steel autoclave. The AAO membrane was then placed vertically in the reaction solution by using a Teflon holder, which ensured that the generated UiO-66-NH_2_ layers were grown on both sides of the AAO membrane. The autoclave reactor was then placed in an oven and heated at 120°C for 1–5 days. After cooling to room temperature, the resulting solution (of a 1-day reaction) was collected for further use. Meanwhile, the single-growth UiO-66-NH_2_ based composite membranes were taken out and washed consecutively three times with ethanol and DMF. This was followed by drying overnight at room temperature.

### Preparation of Secondary-Growth UiO-66-NH_2_ Composite Membrane

The reaction solution collected during the single-growth procedure was transferred into another 50 mL Teflon-lined stainless-steel autoclave for seed growth of UiO-66-NH_2_ MOF on a new AAO support. The autoclave was placed in an oven and heated at 120°C for 24 h. After cooling to room temperature, the old reaction solution was removed, and a new mixture solution (0.116 g ZrCl_4_ and 0.0906 g BDC-NH_2_ in 30 mL of DMF) was transferred into the Teflon container. The autoclave was placed in the oven again and heated at 120°C for 24 h. After cooling to room temperature, the secondary-growth membrane was washed with ethanol and DMF for three times, followed by drying overnight at room temperature.

## Results and Discussion

### Fabrication of UiO-66-NH_2_ Composite Membranes

**FIGURE 1:**
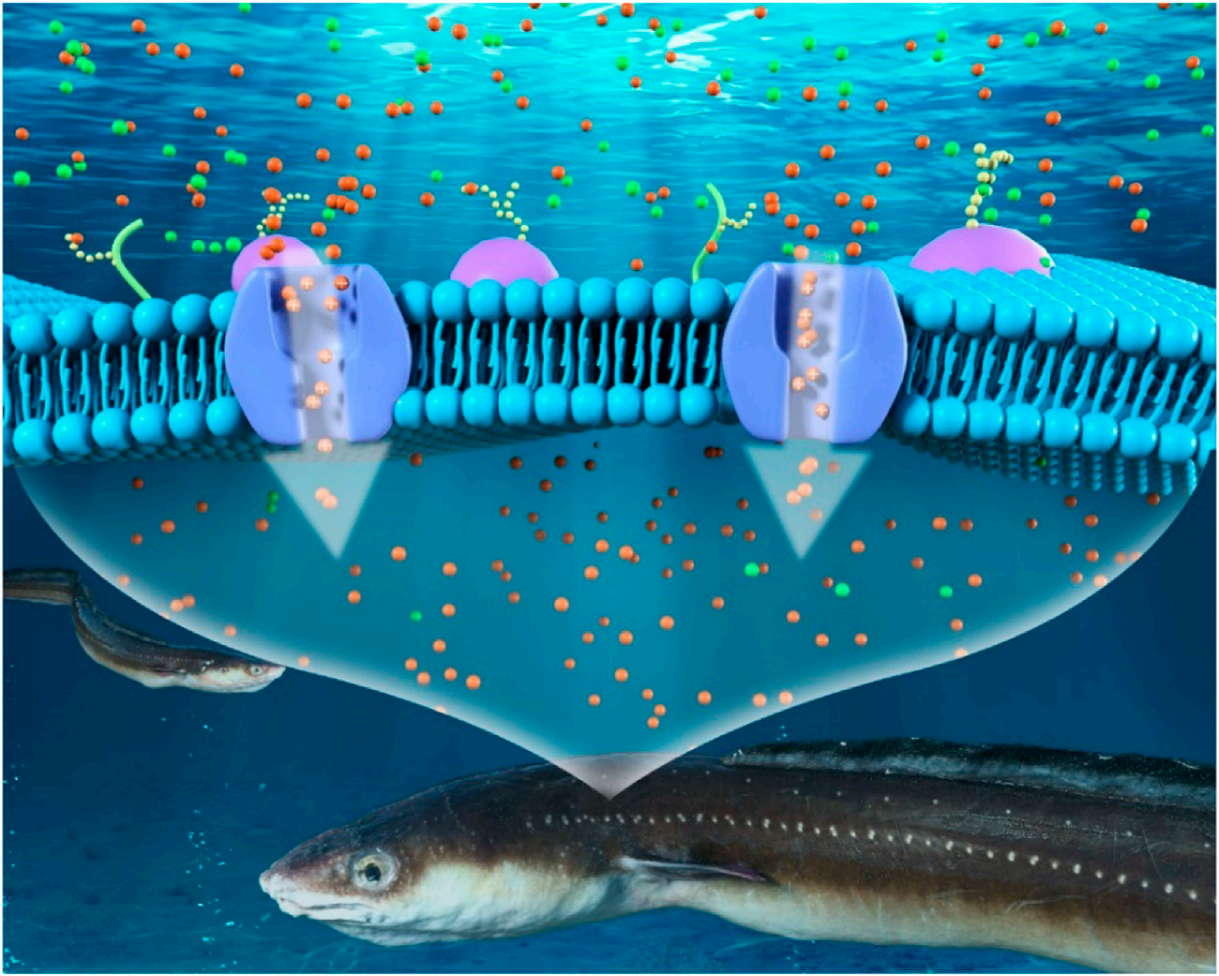
Schematic illustration of the sub-nanometer protein ion channels of an electric ell.

**FIGURE 2:**
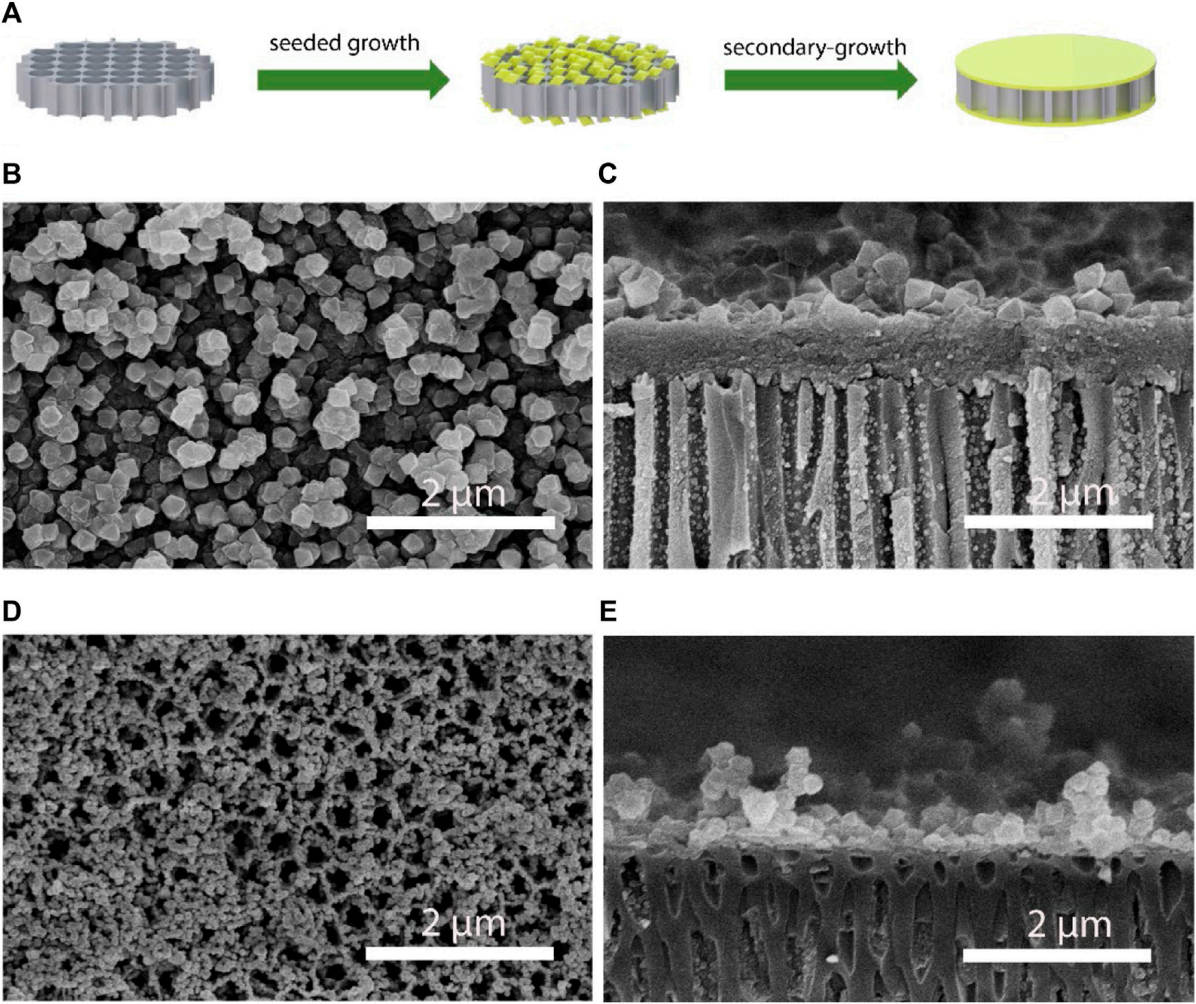
Preparation and characterization of the UiO-66-NH_2_ composite membranes. **(A)** The fabrication process for the UiO-66-NH_2_ composite membranes. Representative **(B)** top and **(C)** cross-sectional SEM images of the secondary-growth UiO-66-NH_2_ membrane. Representative **(D)** top and **(E)** cross-sectional SEM images of the single-growth UiO-66-NH_2_ membrane.

Inspired by the sub-nanometer protein ion-transporting channels of electric ell (Figure 1), we synthesized the UiO-66-NH_2_ MOF based composite membranes for salinity-gradient osmotic energy conversion. The synthesis of the continuous and ultrathin UiO-66-NH_2_ membranes using a seeded secondary-growth method is shown in Figure 2A. In the first step, nm-sized UiO-66-NH_2_ crystals, left in the reaction solution during the single-growth procedure, were used to deposit a seed layer on both surfaces of a porous AAO substrate. The seeded AAO support was then exposed to a UiO-66-NH_2_ precursor solution for secondary growth to form a dense UiO-66-NH_2_ membrane. Scanning electron microscopy (SEM) characterization showed a continuous and dense UiO-66-NH_2_ layer on the AAO support (Figure 2B). The samples exhibited clear octahedral shapes, which suggested high crystallinity. The thickness of the UiO-66-NH_2_ layers was ∼0.71 μm (Figure 2C). Furthermore, it is important to compare the influence of seeds to the single-growth and secondary-growth UiO-66-NH_2_ membranes. The single-growth UiO-66-NH_2_ has a small particle size and numerous defects (Figures 2D,E), while the introduction of a seed layer yields a larger particle size of UiO-66-NH_2_ membranes (Figures 2B,C). In addition, the seeding step was found to be very crucial for forming a continuous MOF membrane. Without a seed layer, a discontinuous and defective layer was observed even when the single-growth period was extended to more than 5 days (Supplementary Figure S1). The X-ray diffraction (XRD) patterns of the UiO-66-NH_2_ crystals were consistent with the reported calculated XRD patterns obtained from simulation (Supplementary Figure S2), confirming the successful synthesis of the UiO-66-NH_2_ MOFs. Moreover, the as-prepared UiO-66-NH_2_ owns a Brunauer–Emmett–Teller surface areas of 520 m^2^/g, sub-1 nanometer size window apertures, and 1.2 nm cavities of MOF channels, as calculated from N_2_ adsorption/desorption isotherm profiles (Supplementary Figure S3). These channel structures were comparable to most hydrated ions in water, therefore proved the potential of UiO-66-NH_2_ membranes for harvesting osmotic energy. The positive framework charge originated from NH_2_ functional groups was demonstrated by the positive zeta potential value, showing the ability of the as-prepared UiO-66-NH_2_ membranes to transport anions selectively (Supplementary Figure S4). The contact angle results show that the composite membrane obtained high hydrophilicity after MOF deposition, and therefore can realize fast and low-resistance fluid transport (Supplementary Figure S5).

### Surface Charge-Governed Ion Transport

**FIGURE 3:**
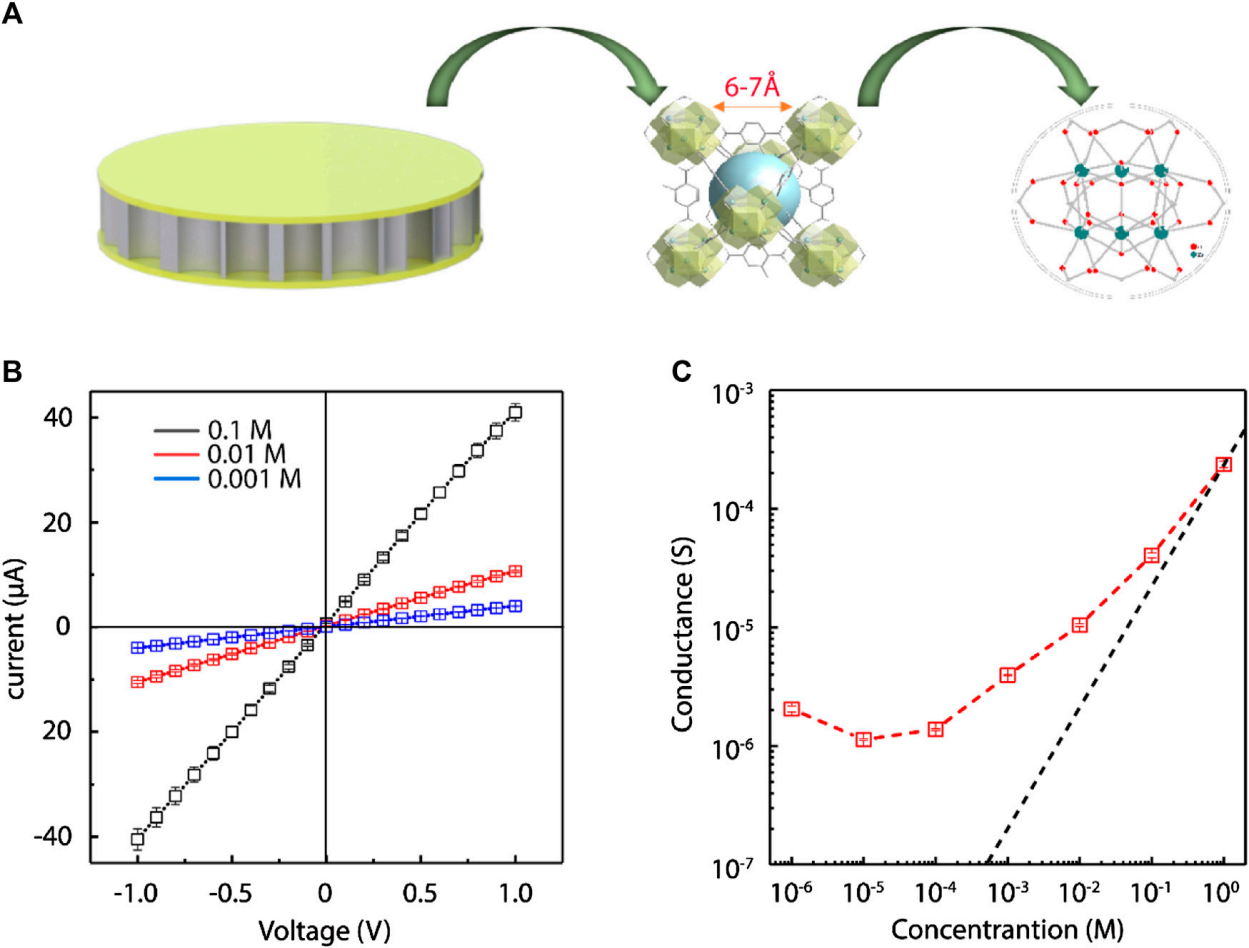
Surface charge-governed ion transport in UiO-66-NH_2_ composite membrane. **(A)** Schematic illustration of the UiO-66-NH_2_ composite membrane with sub-nanometer sized channels. **(B)** Representative *I-V* curves obtained with three different NaCl concentration. **(C)** Ionic conductance of the UiO-66-NH_2_ membranes at different electrolyte concentration. When the salt concentrations were <1 M, the ionic conductance values of the UiO-66-NH_2_ membranes (red square) deviate significantly from the bulk value (black curve), demonstrating the surface charge governed ion transport behavior.

The positively charged framework and sub-1 nanometer apertures of the UiO-66-NH_2_ composite membranes (Figure 3A) suggest that it should have surface charged-governed ion transport at low electrolyte concentration. To demonstrate, we measured the ionic conductivity of the UiO-66-NH_2_ membranes by changing NaCl electrolyte concentration. Two Ag/AgCl electrodes were inserted on either side of the custom-made electrochemical cell (Supplementary Figure S6) to record the current generated by sweeping voltages from −1 V to +1 V. *I–V* curves at different NaCl concentrations were firstly recorded, and *I–V* curves at three representative concentrations are shown in Figure 3B. As MOF layers were deposited on both sides of the AAO support, the ion transport of the composite membrane showed a symmetric behavior. Then, the conductance was calculated from the *I–V* slopes (Figure 3C). At high concentrations, the ionic conductance values of the UiO-66-NH_2_ membranes were similar to that of the bulk phase. However, the ionic conductance started to deviate from the bulk value tendency, and was considerably higher than the bulk value when the salt concentration was below 1 M. When the salt concentration was <0.1 M, the Debye lengths were larger than the window apertures of UiO-66-NH_2_ MOFs. Thus, the anions were the dominant charge carriers, and their concentrations were determined by the surface charge densities of the UiO-66-NH_2_ sub-nanochannels. The result that the conductance of as-prepared composite membrane is larger than that of the bulk phase at low electrolyte concentration demonstrated that ionic transport is controlled by surface charge, which also sheds light on the further application of the as-prepared MOF composite membrane for harvesting salinity-gradient osmotic energy through selective anion transport.

### Evaluation of Electrochemical Properties

**FIGURE 4:**
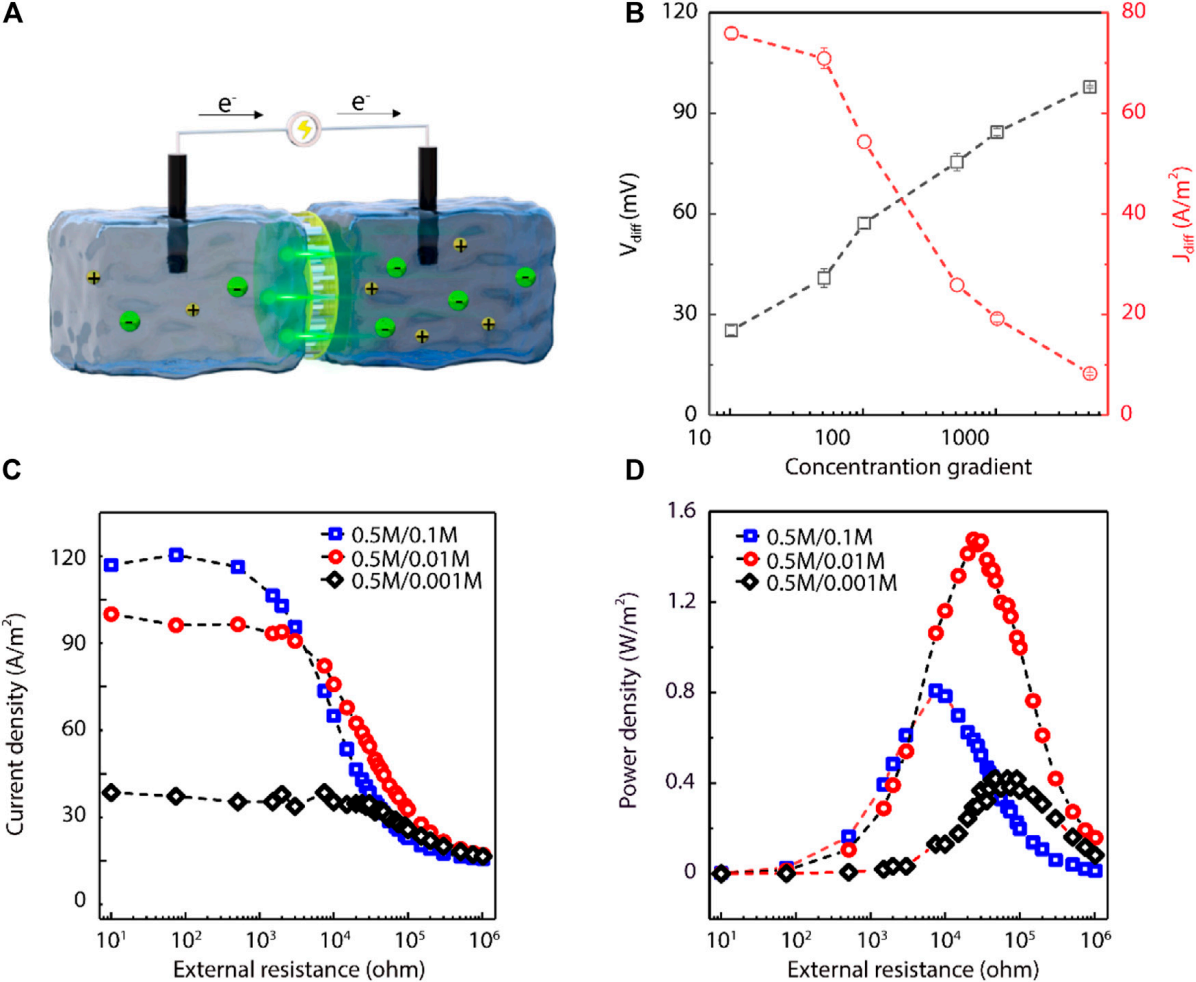
UiO-66-NH_2_ composite membranes for salinity-gradient osmotic energy conversion. **(A)** Schematic illustration of the proposed energy harvesting device. **(B)** As the concentration gradient was increased, the *V*_diff_ gradually increased and the *J*_diff_ gradually decreased. The high NaCl concentration was fixed at 0.5 M. **(C)** Current density and **(D)** power density of the as-prepared UiO-66-NH_2_ composite membranes as a function of the external resistance under three different NaCl concentration gradients. For three salinity gradients, the measured current densities all gradually decrease with increasing external resistance. The maximum power density values were ∼0.8, 1.47, and 0.42 W/m^2^ for 5-, 50-, and 500-fold NaCl concentration gradients, respectively.

The performance of the UiO-66-NH_2_ composite membranes for salinity-gradient osmotic energy conversion was further studied by using asymmetric NaCl electrolyte solutions in the electrochemical cell (Figure 4A). The high concentration solution was standard artificial seawater (0.5 M NaCl), while the low concentration solution ranged from 0.0001 to 0.05 M NaCl. The salinity gradient was converted into electrical energy by the positively charged UiO-66-NH_2_ composite membranes to transport anions selectively. We firstly recorded the current generated by sweeping voltages. The quadrant of the *I-V* curve under a 50-fold NaCl concentration gradient further indicated the positive charge of the UiO-66-NH_2_ MOF (Supplementary Figure S7). During the osmotic energy conversion with asymmetric electrolytes, we eliminated the imbalance in electrode potentials by using a pair of salt bridges. The results showed that the *V*_diff_ values increased as the salinity gradient increased. However, the *J*_diff_ values decreased as the concentration gradient increased (Figure 4B). The reason of *V*_diff_ and *J*_diff_ changing in the opposite ways is that the increase in salinity gradient produces higher osmotic pressure and increases the resistance of the system. In a 50-fold salinity gradient, the *V*_diff_ and *J*_diff_ values of the UiO-66-NH_2_ composite membranes were 40.85 mV and 70.91 A/m^2^, respectively. However, the *V*_diff_ and *J*_diff_ of the single-growth UiO-66-NH_2_ membranes were about 15.69 mV and 70 A/m^2^ respectively (Supplementary Figure S8), which *V*_diff_ was smaller than that of the secondary-growth UiO-66-NH_2_ membranes. In addition, the performance of the single-growth UiO-66-NH_2_ composite membrane did not change obviously with the single-growth period (Supplementary Figure S8), showing the necessity of this seeded secondary-growth procedure for high-performance power-generation UiO-66-NH_2_ composite membrane. Under the 50-fold salinity gradient, the corresponding energy conversion efficiency of the UiO-66-NH_2_ composite membranes was 8.8% (Supplementary Table S1).

The actual power generation performance of the UiO-66-NH_2_ membranes was further investigated by using an external circuit resistor (*R_L_*). *I–t* curves under different external resistances were recorded, where *I* denotes the measured current. Then, the output power density was calculated as *P = I^2^ × R_L_*. Under three salinity gradients, the current density decreased as the external resistance increased because the *V*_diff_ is the same at the same concentration (Figure 4C). However, the output power density increased firstly and then decreased with the increase of resistance, and reached a maximum value when the internal and external resistances were equal (Figure 4D). Moreover, a larger concentration gradient means a comparatively lower concentration on the low salt concentration side, which leads to an increase in the internal resistance of the system. Due to that, the power density of 500-fold salinity gradient turned smaller than that of the 50-fold salinity gradient. The resultant power density values were 0.80, 1.47, and 0.42 W/m^2^ under 5-, 50-, and 500-fold NaCl gradients, respectively, indicating the practical application merits in estuaries with different concentration gradients. In contrast, the power density of the AAO support itself at the 50-fold NaCl concentration gradient was only 0.07 W/m^2^, much less than that of the UiO-66-NH_2_ composite membrane (Supplementary Figure S9), further indicating that the power generation performance of the composite membrane was ascribed to the MOF layer with sub-nanometer channels. It is worth noting that the power density measured here is not low compared with other MOF based materials (Supplementary Table S2).

Membranes with another two different thickness of MOF layer have also been fabricated (Supplementary Figure S10), to investigate the influence of MOF thickness to energy conversion. The results demonstrated that under the 50-fold NaCl concentration gradient, the power density values decreased from 1.47 to 0.42 W/m^2^ due to the increased internal resistance caused by the increase in MOF layer thickness (Supplementary Figure S11). Furthermore, to prove that the UiO-66-NH_2_ membranes can be applied to different fields, we measured the current density and power density of the UiO-66-NH_2_ membranes at different pH conditions (Supplementary Figure S12). Under the 50-fold NaCl concentration gradient, the power density values decreased from 3.2 to 1.2 W/m^2^ when the pH values of the solutions increased from 2 to 11. This is because of that positively charged materials have a higher surface charge density under more acidic condition, therefore exhibiting better ion-transport performance and higher power generation capability. The related zeta potential results also proved this explanation (Supplementary Figure S13). The external resistance values corresponding to the maximum value of power densities were close even under different pH conditions, indicating that the internal resistance was independent of pH at the same concentration gradient. These results demonstrated that the ion transport behaviour of the UiO-66-NH_2_ membranes can be modulated by adjusting pH values, and therefore high performance of salinity-gradient osmotic energy generation can be achieved by further tailoring the surface charge properties of UiO-66-NH_2_ MOF, as we expected.

### Stability of the UiO-66-NH_2_ Composite Membranes

**FIGURE 5:**
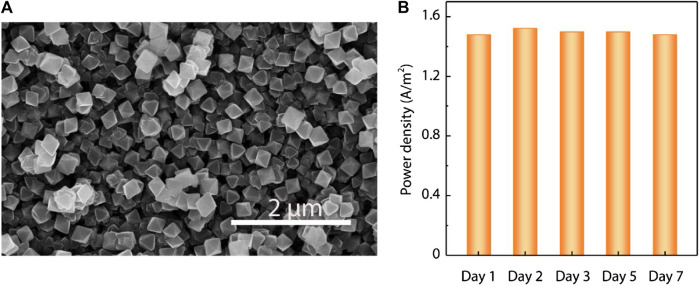
Long-term stability of the as-prepared UiO-66-NH_2_ composite membranes. **(A)** Representative SEM images of the UiO-66-NH_2_ composite membranes, showing no obvious change after immersion in deionized water for 1 month. **(B)** Under 50-fold NaCl concentration gradient, the output power density of the UiO-66-NH_2_ composite membranes showed strong stability examined in 1 week.

More importantly, we demonstrated the long-term stability of the proposed UiO-66-NH_2_ composite membranes. The UiO-66-NH_2_ membranes maintained their continuous and dense structures after immersion in deionized water for 1 month (Figure 5A; Supplementary Figure S14). XRD patterns also indicate that there is no obvious change of the MOF crystal structure (Supplementary Figure S15). The long-term power generation stability of the UiO-66-NH_2_ membrane was also confirmed, as the output power density was found maintained for at least 1 week (Figure 5B). This further suggests the good application viability of the MOF membranes in practical osmotic energy harvesting.

## Conclusion

In summary, we prepared positively charged UiO-66-NH_2_ MOF based composite membrane with sub-1-nm windows through a simple seed-assisted secondary growth method, and achieved successful capture of salinity-gradient osmotic energy by anion-selective ion transport. The secondary-growth MOF membrane showed better structure and properties than the single-growth membrane. The as-prepared UiO-66-NH_2_ composite membrane was also practical based on the results of power density under different salinity gradients and pH conditions and their long-term stability of structure and performance. We note that by adjusting the physical structures and the surface chemistry of the composite membrane, the power generation capability can be further improved. The current work suggests the potential of the UiO-66-NH_2_ composite membrane in various practical applications, and provides inspirations for designing anion-selective and sub-nanochannel based membranes towards high-performance osmotic energy harvesting.

## Data Availability

The original contributions presented in the study are included in the article/Supplementary Material, further inquiries can be directed to the corresponding authors.

## References

[B1] Cai P. Xu M. Meng S. S. Lin Z. Yan T. Drake H. F. (2021). Precise Spatial‐Designed Metal‐Organic‐Framework Nanosheets for Efficient Energy Transfer and Photocatalysis. Angew. Chem. Int. Ed. 60 (52), 27258–27263. 10.1002/anie.202111594 34714946

[B2] Ding L. Xiao D. Lu Z. Deng J. Wei Y. Caro J. (2020). Oppositely Charged Ti 3 C 2 T X MXene Membranes with 2D Nanofluidic Channels for Osmotic Energy Harvesting. Angew. Chem. Int. Ed. 59 (22), 8720–8726. 10.1002/anie.201915993 31950586

[B3] Gao J. Feng Y. Guo W. Jiang L. (2017). Nanofluidics in Two-Dimensional Layered Materials: Inspirations from Nature. Chem. Soc. Rev. 46 (17), 5400–5424. 10.1039/c7cs00369b 28722059

[B4] Gao J. Guo W. Feng D. Wang H. Zhao D. Jiang L. (2014). High-Performance Ionic Diode Membrane for Salinity Gradient Power Generation. J. Am. Chem. Soc. 136 (35), 12265–12272. 10.1021/ja503692z 25137214

[B5] Gao J. Liu X. Jiang Y. Ding L. Jiang L. Guo W. (2019). Understanding the Giant Gap between Single‐Pore‐ and Membrane‐Based Nanofluidic Osmotic Power Generators. Small 15 (11), 1804279. 10.1002/smll.201804279 30653272

[B6] Jiang Y. Ma W. Qiao Y. Xue Y. Lu J. Gao J. (2020). Metal-Organic Framework Membrane Nanopores as Biomimetic Photoresponsive Ion Channels and Photodriven Ion Pumps. Angew. Chem. Int. Ed. 59 (31), 12795–12799. 10.1002/anie.202005084 32343466

[B7] Kadhom M. Deng B. (2018). Metal-Organic Frameworks (MOFs) in Water Filtration Membranes for Desalination and Other Applications. Appl. Mater. Today 11, 219–230. 10.1016/j.apmt.2018.02.008

[B8] Kirchon A. Feng L. Drake H. F. Joseph E. A. Zhou H.-C. (2018). From Fundamentals to Applications: A Toolbox for Robust and Multifunctional MOF Materials. Chem. Soc. Rev. 47 (23), 8611–8638. 10.1039/c8cs00688a 30234863

[B9] Lao J. Lv R. Gao J. Wang A. Wu J. Luo J. (2018). Aqueous Stable Ti3C2 MXene Membrane with Fast and Photoswitchable Nanofluidic Transport. ACS Nano 12 (12), 12464–12471. 10.1021/acsnano.8b06708 30495925

[B10] Lao J. Wu S. Gao J. Dong A. Li G. Luo J. (2020). Electricity Generation Based on a Photothermally Driven Ti3C2Tx MXene Nanofluidic Water Pump. Nano Energy 70, 104481. 10.1016/j.nanoen.2020.104481

[B11] Li X. Zhang H. Wang P. Hou J. Lu J. Easton C. D. (2019). Fast and Selective Fluoride Ion Conduction in Sub-1-nanometer Metal-Organic Framework Channels. Nat. Commun. 10, 2490. 10.1038/s41467-019-10420-9 31186413 PMC6560108

[B12] Liu X. He M. Calvani D. Qi H. Gupta K. B. S. S. de Groot H. J. M. (2020). Power Generation by Reverse Electrodialysis in A Single-Layer Nanoporous Membrane Made from Core-Rim Polycyclic Aromatic Hydrocarbons. Nat. Nanotechnol. 15 (4), 307–312. 10.1038/s41565-020-0641-5 32152558

[B13] Liu Y.-C. Yeh L.-H. Zheng M.-J. Wu K. C.-W. (2021). Highly Selective and High-Performance Osmotic Power Generators in Subnanochannel Membranes Enabled by Metal-Organic Frameworks. Sci. Adv. 7 (10), eabe9924. 10.1126/sciadv.abe9924 33658204 PMC7929511

[B14] Lu J. Zhang H. Hu X. Qian B. Hou J. Han L. (2021). Ultraselective Monovalent Metal Ion Conduction in a Three-Dimensional Sub-1 Nm Nanofluidic Device Constructed by Metal-Organic Frameworks. ACS Nano 15 (1), 1240–1249. 10.1021/acsnano.0c08328 33332960

[B15] Man Z. Safaei J. Zhang Z. Wang Y. Zhou D. Li P. (2021). Serosa-Mimetic Nanoarchitecture Membranes for Highly Efficient Osmotic Energy Generation. J. Am. Chem. Soc. 143 (39), 16206–16216. 10.1021/jacs.1c07392 34570466

[B16] Montenegro J. Ghadiri M. R. Granja J. R. (2013). Ion Channel Models Based on Self-Assembling Cyclic Peptide Nanotubes. Acc. Chem. Res. 46 (12), 2955–2965. 10.1021/ar400061d 23898935 PMC3867521

[B17] Ren C. Chen F. Ye R. Ong Y. S. Lu H. Lee S. S. (2019). Molecular Swings as Highly Active Ion Transporters. Angew. Chem. Int. Ed. 58 (24), 8034–8038. 10.1002/anie.201901833 30983075

[B18] Rice A. M. Leith G. A. Ejegbavwo O. A. Dolgopolova E. A. Shustova N. B. (2019). Heterometallic Metal-Organic Frameworks (MOFs): The Advent of Improving the Energy Landscape. ACS Energ. Lett. 4 (8), 1938–1946. 10.1021/acsenergylett.9b00874

[B19] Ruan H. Pan N. Wang C. Yu L. Liao J. Shen J. (2021). Functional UiO-66 Series Membranes with High Perm Selectivity of Monovalent and Bivalent Anions for Electrodialysis Applications. Ind. Eng. Chem. Res. 60 (10), 4086–4096. 10.1021/acs.iecr.0c05992

[B20] Sadeghi G. (2022). Energy Storage on Demand: Thermal Energy Storage Development, Materials, Design, and Integration Challenges. Energ. Storage Mater. 46, 192–222. 10.1016/j.ensm.2022.01.017

[B21] Schroeder T. B. H. Guha A. Lamoureux A. VanRenterghem G. Sept D. Shtein M. (2017). An Electric-Eel-Inspired Soft Power Source from Stacked Hydrogels. Nature 552, 214–218. 10.1038/nature24670 29239354 PMC6436395

[B22] Siria A. Bocquet M.-L. Bocquet L. (2017). New Avenues for the Large-Scale Harvesting of Blue Energy. Nat. Rev. Chem. 1 (11), 0091. 10.1038/s41570-017-0091

[B23] Sun Y. Dong T. Lu C. Xin W. Yang L. Liu P. (2020). Tailoring A Poly(ether Sulfone) Bipolar Membrane: Osmotic‐Energy Generator with High Power Density. Angew. Chem. Int. Ed. 59 (40), 17423–17428. 10.1002/anie.202006320 32578316

[B24] Tan H. Zhou Y. Qiao S.-Z. Fan H. J. (2021). Metal Organic Framework (MOF) in Aqueous Energy Devices. Mater. Today 48, 270–284. 10.1016/j.mattod.2021.03.011

[B25] Tawalbeh M. Al-Othman A. Abdelwahab N. Alami A. H. Olabi A. G. (2021). Recent Developments in Pressure Retarded Osmosis for Desalination and Power Generation. Renew. Sustainable Energ. Rev. 138, 110492. 10.1016/j.rser.2020.110492

[B26] Teng Y. Kong X.-Y. Liu P. Qian Y. Hu Y. Fu L. (2021). A Universal Functionalization Strategy for Biomimetic Nanochannel via External Electric Field Assisted Non-covalent Interaction. Nano Res. 14 (5), 1421–1428. 10.1007/s12274-020-3192-z

[B27] van Ruijven B. J. De Cian E. Sue Wing I. (2019). Amplification of Future Energy Demand Growth Due to Climate Change. Nat. Commun. 10, 2762. 10.1038/s41467-019-10399-3 31235700 PMC6591298

[B28] Wan L. Zhou C. Xu K. Feng B. Huang A. (2017). Synthesis of Highly Stable UiO-66-NH2 Membranes with High Ions Rejection for Seawater Desalination. Microporous Mesoporous Mater. 252, 207–213. 10.1016/j.micromeso.2017.06.025

[B29] Xiao K. Jiang L. Antonietti M. (2019). Ion Transport in Nanofluidic Devices for Energy Harvesting. Joule 3 (10), 2364–2380. 10.1016/j.joule.2019.09.005

[B30] Xin W. Xiao H. Kong X.-Y. Chen J. Yang L. Niu B. (2020). Biomimetic Nacre-like Silk-Crosslinked Membranes for Osmotic Energy Harvesting. ACS Nano 14 (8), 9701–9710. 10.1021/acsnano.0c01309 32687698

[B31] Xin W. Zhang Z. Huang X. Hu Y. Zhou T. Zhu C. (2019). High-Performance Silk-Based Hybrid Membranes Employed for Osmotic Energy Conversion. Nat. Commun. 10, 3876. 10.1038/s41467-019-11792-8 31462636 PMC6713777

[B32] Yang L. Liu P. Zhu C. Zhao Y. Yuan M. Kong X.-Y. (2021). Ion Transport Regulation through Triblock Copolymer/PET Asymmetric Nanochannel Membrane: Model System Establishment and Rectification Mapping. Chin. Chem. Lett. 32 (2), 822–825. 10.1016/j.cclet.2020.04.047

[B33] Yip N. Y. Brogioli D. Hamelers H. V. M. Nijmeijer K. (2016). Salinity Gradients for Sustainable Energy: Primer, Progress, and Prospects. Environ. Sci. Technol. 50 (22), 12072–12094. 10.1021/acs.est.6b03448 27718544

[B34] Zhang Z. Kong X.-Y. Xiao K. Liu Q. Xie G. Li P. (2015). Engineered Asymmetric Heterogeneous Membrane: A Concentration-Gradient-Driven Energy Harvesting Device. J. Am. Chem. Soc. 137 (46), 14765–14772. 10.1021/jacs.5b09918 26535954

[B35] Zhang Z. Sui X. Li P. Xie G. Kong X.-Y. Xiao K. (2017). Ultrathin and Ion-Selective Janus Membranes for High-Performance Osmotic Energy Conversion. J. Am. Chem. Soc. 139 (26), 8905–8914. 10.1021/jacs.7b02794 28602079

[B36] Zhang Z. Yang S. Zhang P. Zhang J. Chen G. Feng X. (2019). Mechanically Strong MXene/Kevlar Nanofiber Composite Membranes as High-Performance Nanofluidic Osmotic Power Generators. Nat. Commun. 10, 2920. 10.1038/s41467-019-10885-8 31266937 PMC6606750

[B37] Zhao F. Cheng H. Zhang Z. Jiang L. Qu L. (2015). Direct Power Generation from a Graphene Oxide Film under Moisture. Adv. Mater. 27 (29), 4351–4357. 10.1002/adma.201501867 26088604

[B38] Zhao Y. Wei Y. Lyu L. Hou Q. Caro J. Wang H. (2020). Flexible Polypropylene-Supported ZIF-8 Membranes for Highly Efficient Propene/Propane Separation. J. Am. Chem. Soc. 142 (50), 20915–20919. 10.1021/jacs.0c07481 33270450

