# Supplementary material for: Bio-Inspired Salinity-Gradient Power Generation With UiO-66-NH2 Metal-Organic Framework Based Composite Membrane
Source: Front Bioeng Biotechnol. 2022 Apr 21;10:901507. doi: 10.3389/fbioe.2022.901507 (PMC9068881; doi:10.3389/fbioe.2022.901507)
Supplement: Supplementary file 1 [file DataSheet1.docx]

***Supplementary Material***

# Characterization

SEM images of the as-prepared UiO-66-NH_2_ composite membranes were taken using field-emission scanning electron microscopes (FE-SEM, S4800, Hitachi, Japan) operating at an accelerating voltage of 10 kV (for top and bottom views) or 5 kV (for cross-sectional view). BET surface area and pore size distribution were determined by measuring N_2_ adsorption/desorption isotherms at 77 K using an automatic instrument (ASAP2020, Micromeritics Inc., USA). XRD patterns were measured in the 2θ degree varying from 5°- 40° at room temperature using Rigaku X-ray diffractometer (Bruke, D8 advance) with Cu Kα radiation (λ=1.5418 Å , 40 kV and 40 mA) at a scan rate of 10°/min with a step of 0.02°. The zeta potential of the MOF particles was conducted using Zetasizer Nano ZS (Malvern Instruments, UK) in 0.01 M KCl solution.

# Electrical measurement

The UiO-66-NH_2_ composite membrane were mounted between a ‘H’ type electrochemical cell. The electrical measurements were carried out with a Keithley 2450 SourceMeter. A pair of Ag/AgCl electrodes was used. In addition, a pair of salt bridge was used to eliminate the imbalanced electric potential. The *I-V* results were recorded by changing the voltage between -1 and +1 with 0.02 V steps, and the transmembrane conductance was calculated at -1 V. The effective experimental area of the UiO-66-NH_2_ composite membrane was 0.03 mm^2^ during electrical measurement.

# Energy conversion efficiency

The UiO-66-NH_2_ membranes are positively charged in neutral electrolyte solution and possesses anion selectivity. Therefore, the transference number of anions (t^-^) of the membrane under various concentration gradients can be calculated by

$$2t^{-}=\frac{V_{diff}}{(\frac{RT}{F})\ln\frac{{}_{H}C_{H}}{{}_{L}C_{L}}}+1$$

Here, V_diff_ is the diffusion potential; R, T, and F are the gas constant, absolute temperature, and Faraday constant, respectively; γ is the activity coefficient of salt solutions. The subscripts, H and L, denote the properties of salt solutions in high and low salinity reservoirs, respectively. t^-^=1 indicates the membrane with an idea complete anion-selectivity and the more deviation of t^-^ from 0.5 to 1 the more apparent anion selectivity. Upon obtaining t^-^, the energy conversion efficiency (η_max_) corresponding to the maximum power can be determined by

$${}_{max}=\frac{{(2t^{-}-1)}^{2}}{2}\times100\%$$

The transference number of anions as well as conversion efficiency of the UiO-66-NH_2_ membranes as a function of the NaCl gradient were summarized in Table S1.


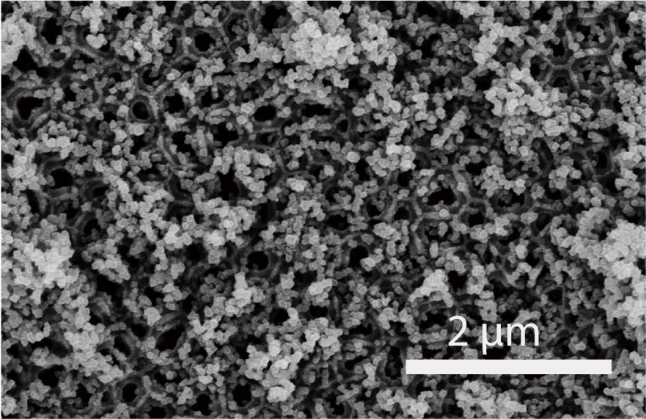


**Supplementary Figure 1. Top-view SEM image of single-growth UiO-66-NH_2_ membrane** with a growth period extended to 120 h.


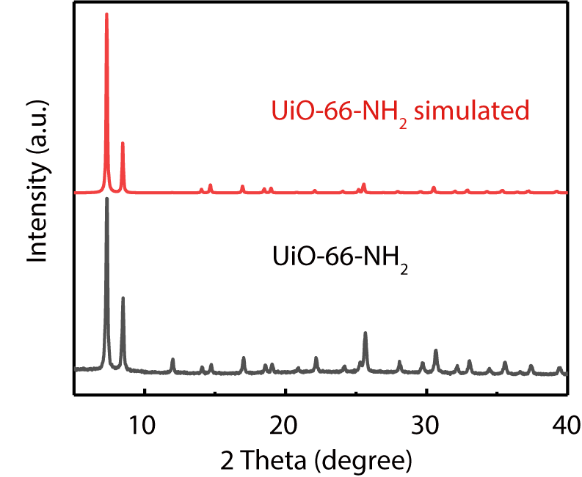


**Supplementary Figure 2. XRD pattern of the as-prepared UiO-66-NH_2_ MOFs.** The result indicates that the UiO-66-NH_2_ layer has been successfully synthesized onto the AAO supporter.


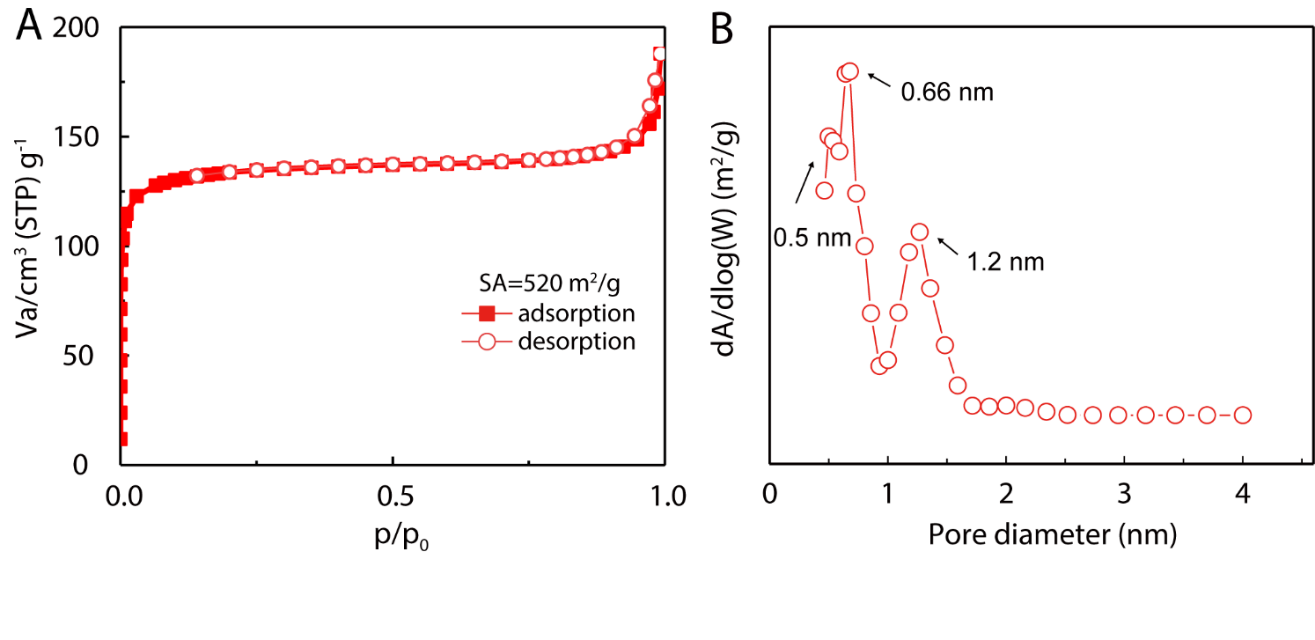


**Supplementary Figure 3.** **N_2_ adsorption/desorption isotherms of UiO-66-NH_2_ MOFs** (**A**) The specific surface area (SA) of UiO-66-NH_2_ MOFs was calculated based on the BET (Brunauer-Emmett-Teller) theory. (**B**) Nominal pore size distributions of UiO-66-NH_2_ MOFs calculated based on the N_2_ adsorption/desorption isotherms by using the nonlocal density functional theory model.


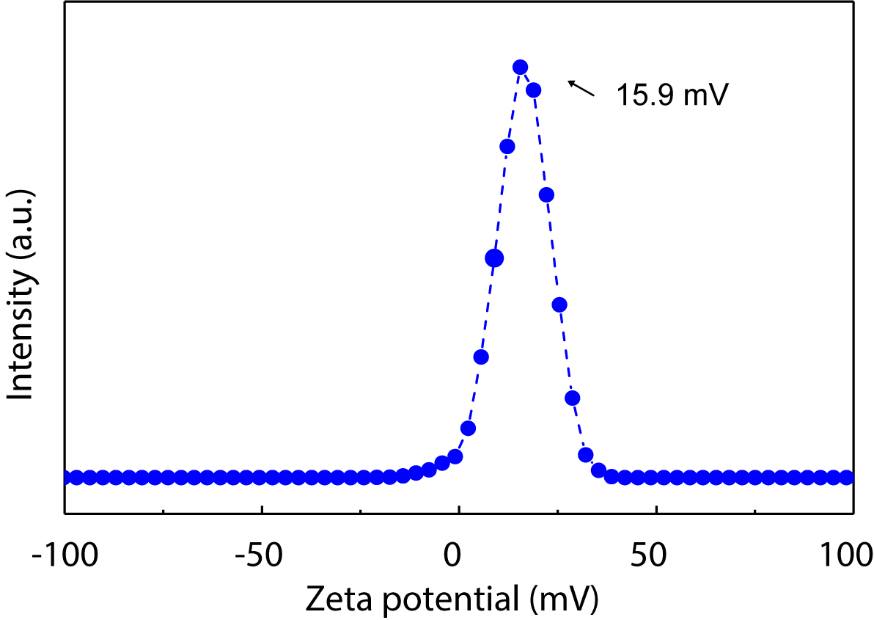


**Supplementary Figure 4. Zeta potential of UiO-66-NH_2_ MOFs.** The measurement was performed in 10 mM KCl.


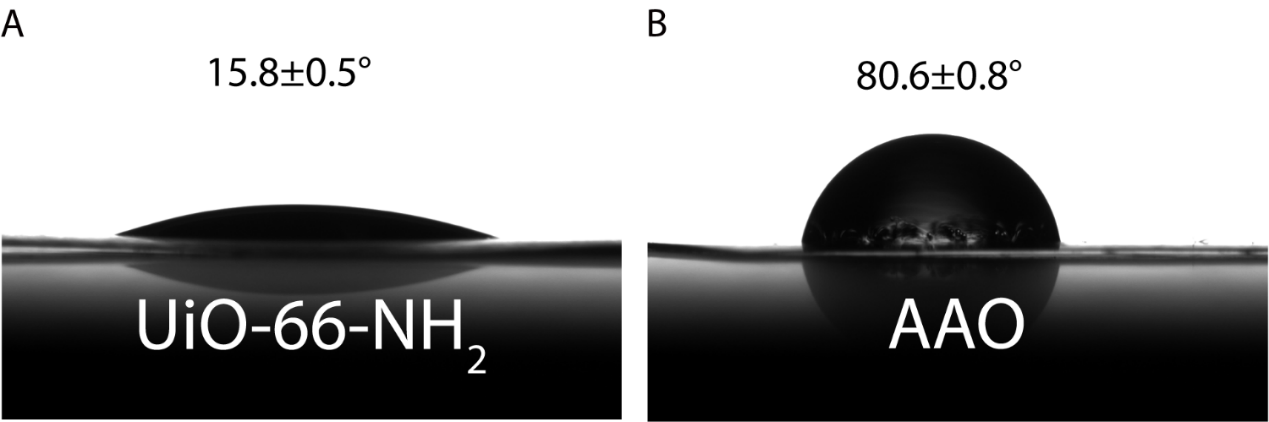


**Supplementary Figure 5. Contact angle measurements of the UiO-66-NH_2_ composite membrane and the original AAO supporter.**


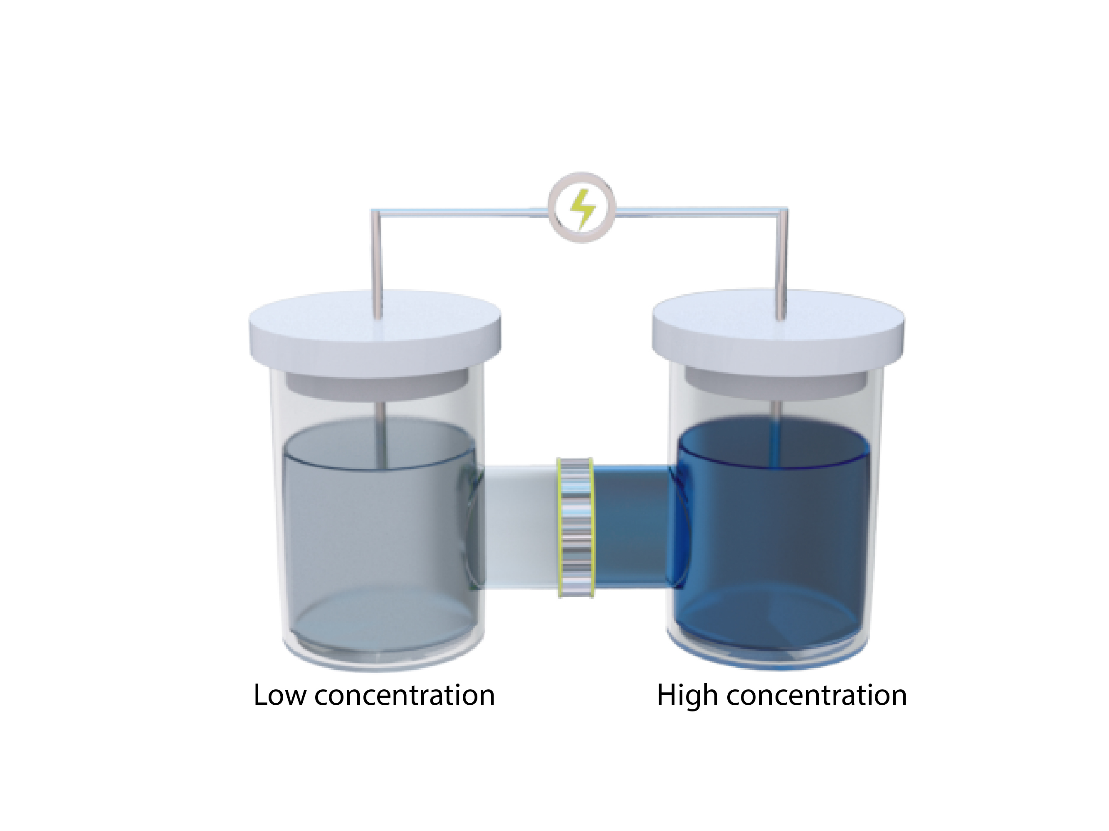


**Supplementary Figure 6. Schematic illustration of the energy harvesting device.**


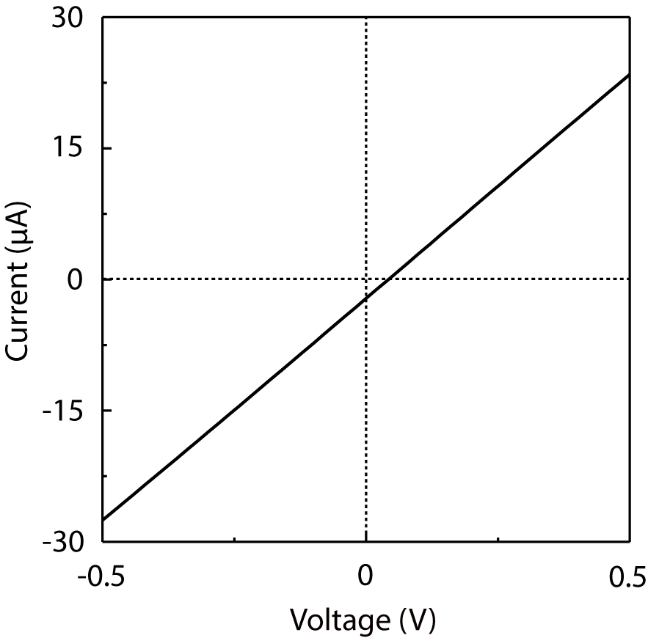


**Supplementary Figure 7. *I-V* curve of the UiO-66-NH_2_ composite membrane recorded under 50-fold NaCl (0.5M/0.01M) gradient.** Positive value of open circuit and negative value of short circuit current indicate that the current is generated by anionic transport, which further proves the positive charge of UiO-66-NH_2_ MOFs.


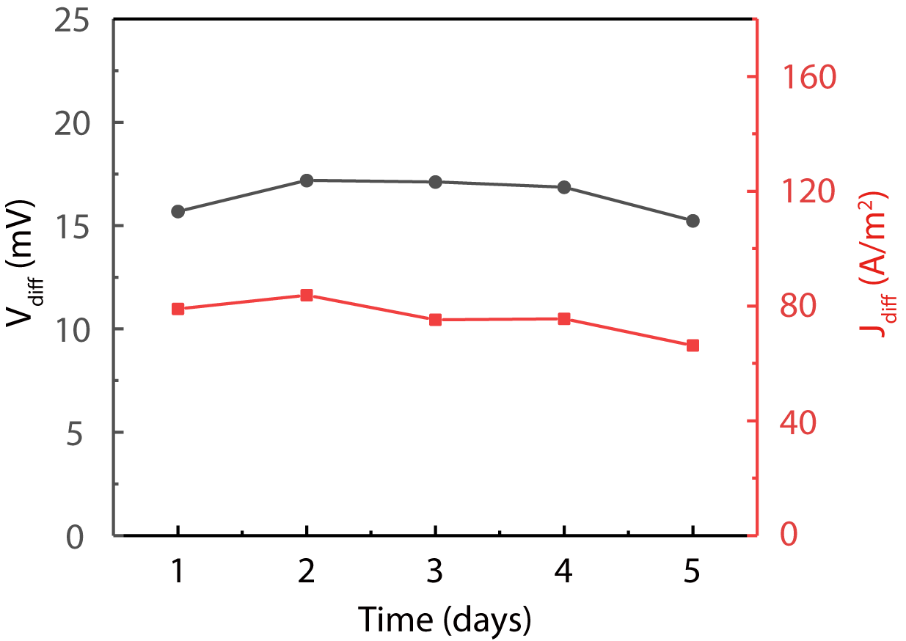


**Supplementary Figure 8. The V_diff_ and J_diff_ of the single-growth UiO-66-NH_2_ composite membranes**, measured at the 50-fold NaCl (0.5M/0.01M) gradient, were independent of the single-growth period.


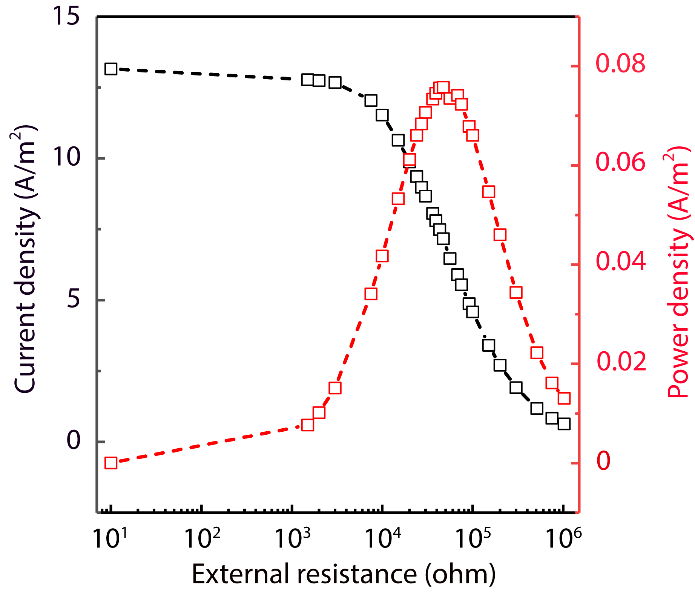


**Supplementary Figure 9. Salinity-gradient osmotic energy conversion of the AAO support.** The maximum output power density of the AAO support was 0.07 W/m^2^.


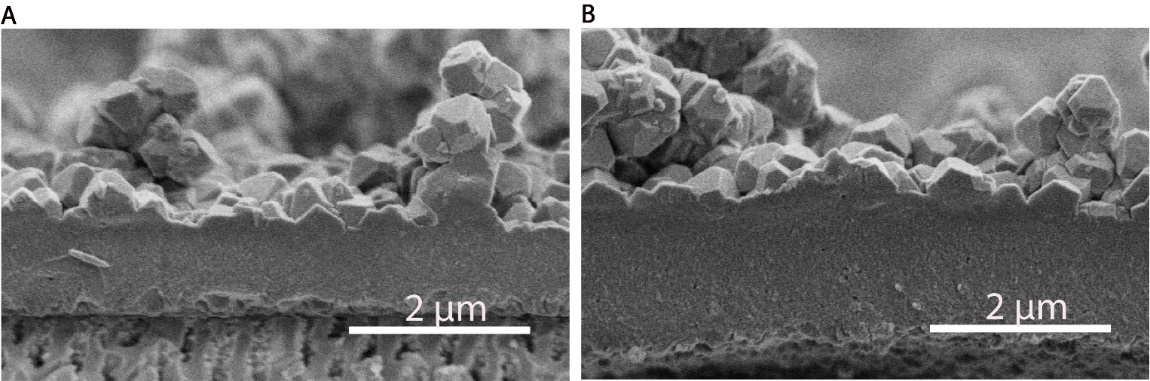


**Supplementary Figure 10. Cross-sectional SEM images of the UiO-66-NH_2_ membranes with different thickness (1.1 µm and 1.9 µm).**


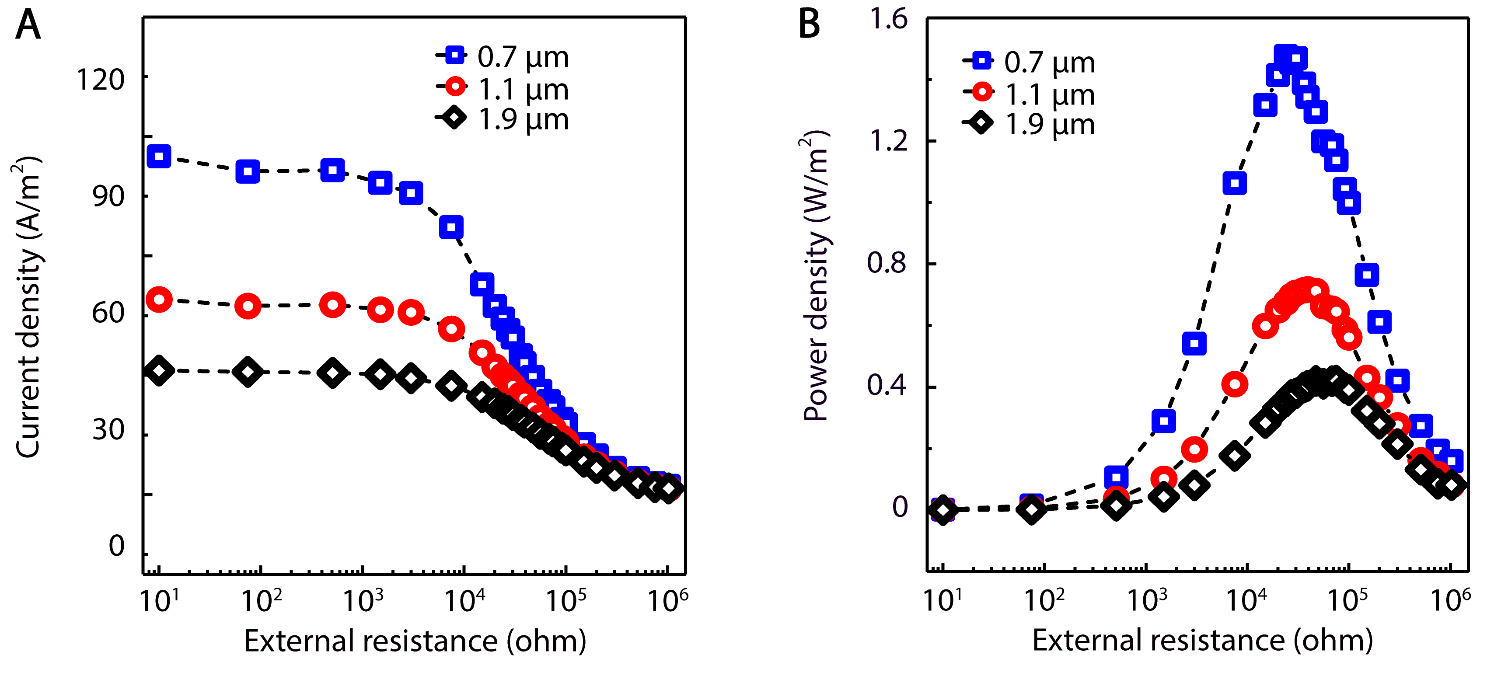


**Supplementary Figure 11. Current density and power density of the UiO-66-NH_2_ membranes with different thickness.** Due to the increased internal resistance caused by increased membrane thickness, both current density and power density decrease with the increase of membrane thickness. The maximum output power density was 1.47, 0.71 and 0.42 W/m^2^ for membranes with thickness of 0.7 μm, 1.1 μm and 1.9 μm.


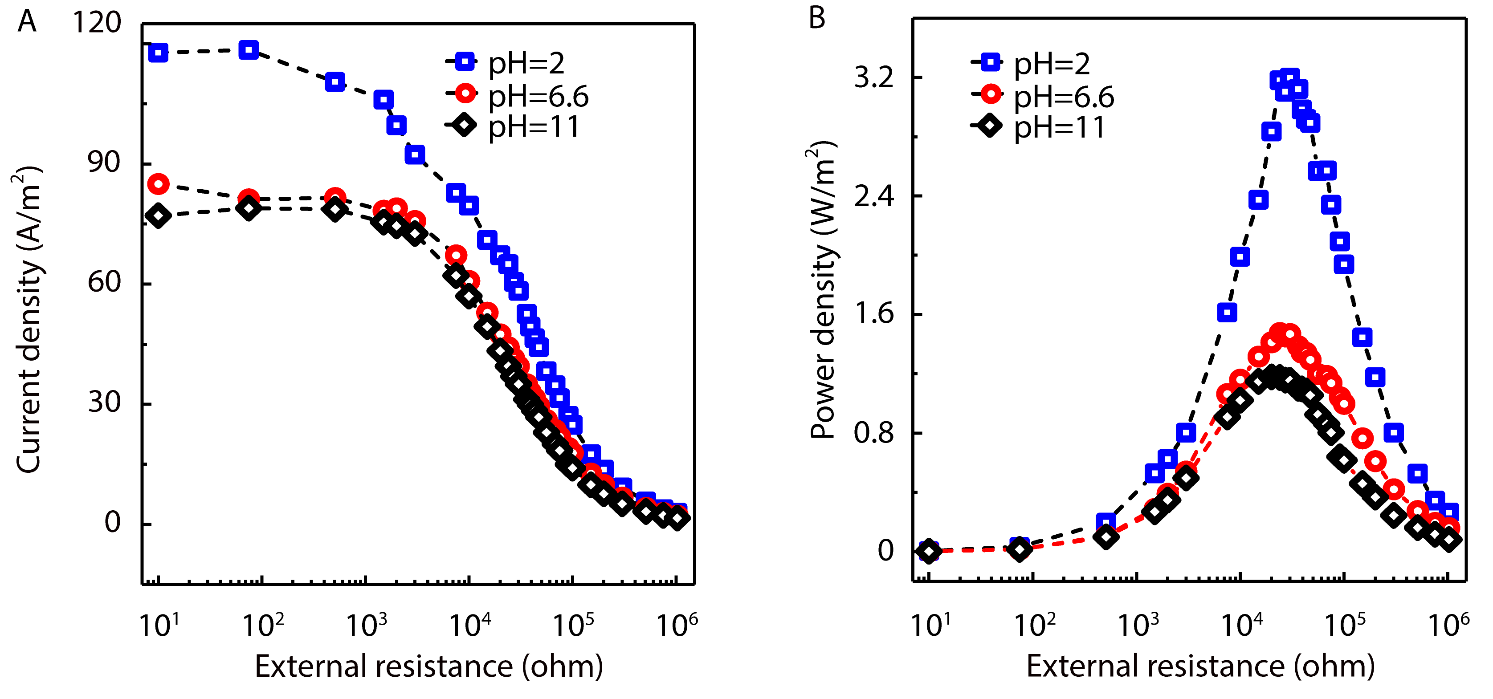


**Supplementary Figure 12. Salinity-gradient osmotic energy conversion of the UiO-66-NH_2_ composite membranes at different pH condition.** Current density (A) and power density (B) harvested at different pH condition. The maximum output power density achieved was ~3.2, 1.47, and 1.17 W/m^2^ at pH=2, 6.6, and 11, respectively.

**
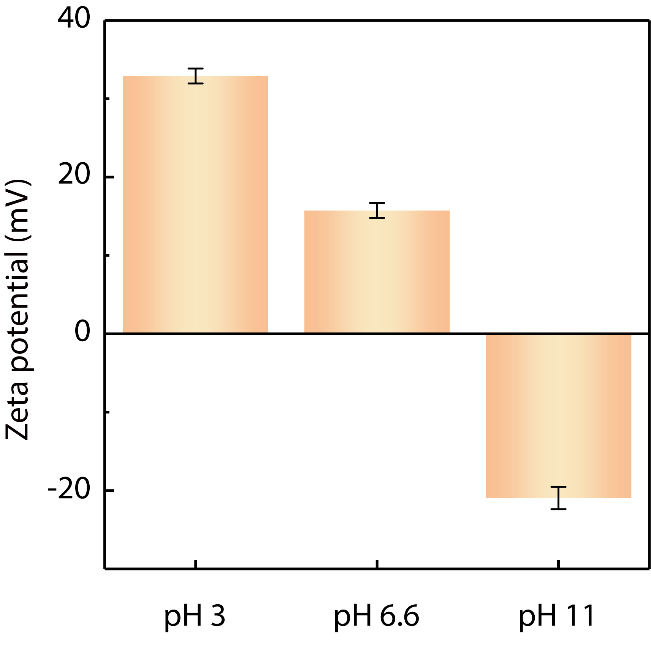
**

**Supplementary Figure 13. Zeta potential of UiO-66-NH_2_ MOFs.** The measurement was performed in 10 mM KCl.

**
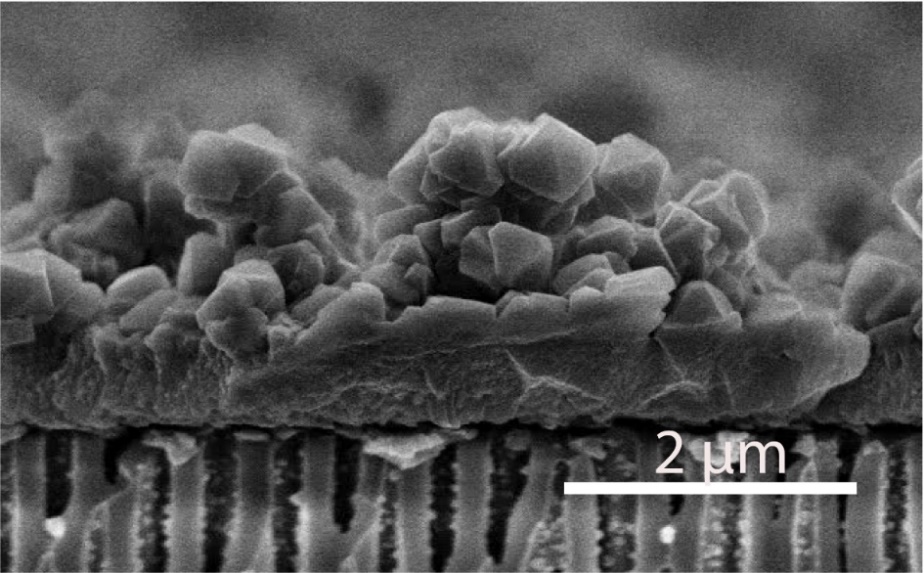
**

**Supplementary Figure 14. Cross-sectional SEM image of the UiO-66-NH_2_ membrane after immersion in deionized water for 1 month,** further indicating the structural stability of the membrane from the morphology side.


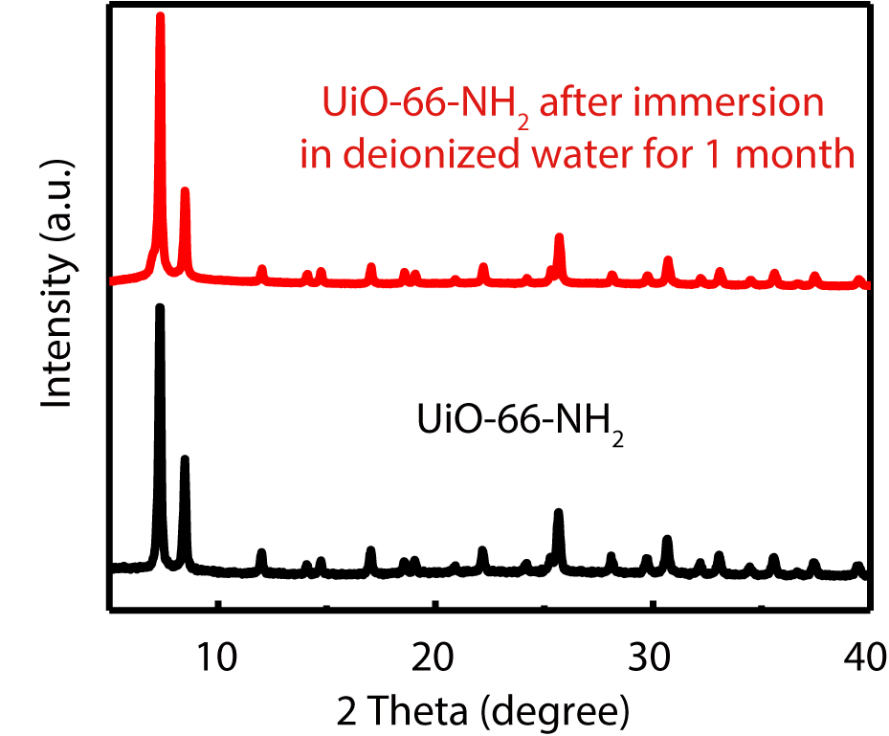


**Supplementary Figure 15. XRD patterns of UiO-66-NH2 membrane before and after immersion in deionized water for 1 month.** No obvious changes were detected, proving the structural stability of MOF crystal.

**Table S1. V_diff_, transference number of anions (t^-^), and maximum conversion efficiency (𝜂_max_) of the UiO-66-NH_2_ composite membrane.**

| Concentration gradient  (M/M) | V_diff_  (mV) | t ^−^ | 𝜂_max_  (%) |
| --- | --- | --- | --- |
| 0.5/0.05 | 25.27 | 0.73 | 10.6 |
| 0.5/0.01 | 40.85 | 0.71 | 8.8 |
| 0.5/0.005 | 57.18 | 0.75 | 12.5 |
| 0.5/0.001 | 75.43 | 0.74 | 11.5 |
| 0.5/0.0005 | 84.36 | 0.75 | 12.5 |
| 0.5/0.0001 | 97.85 | 0.73 | 10.6 |

**Table S2. Comparison of power density with other MOF based materials previously reported.**

| Materials | Salt Type, Concentration ratio (C_H_/C_L_) | | Power density (W/m^-2^) | Refs. |
| --- | --- | --- | --- | --- |
| UiO-66-NH_2_/AAO (+) | NaCl, 0.5 M/0.01 M | 1.47 | | This work |
| polymer/MOF/AAO (-) | KCl, 0.1 M/1 mM | 2.87 | | [1] |
| ZIF_hep_/AAO (-) | KCl, 0.1 M/1 μM | 0.115 | | [2] |
| UiO-66-NH_2_/AAO (+) | KCl, 0.5 M/0.01 M | 4.93 | | [3] |
| PSS/MOF-199 (-) | NaCl, 0.5 M/0.01 M | 2.87 | | [4] |
| Cu-TCPP(Fe) MOFs/PAA (-) | KCl, 1 M/0.001 M | 1.6 | | [5] |

[1] Angew. Chem. Int. Ed. 2022, e202202698.

[2] ACS Appl. Mater. Interfaces 2019, 11, 35496-35500.

[3] Sci. Adv. 2021, 7, eabe9924.

[4] Nano Energy 2018, 53, 643-649.

[5] Adv. Funct. Mater. 2020, 30, 1908804.
